# Twenty-First Annual Meeting of the American Pharmaceutical Association

**Published:** 1873

**Authors:** 


### THE TWENTY-FIRST ANNUAL MEETING OF THE AMERICAN PHARMACEUTICAL ASSOCIATION.

This meeting commenced on the afternoon of Thursday, September 16th, in the Virginia Opera House, located on Ninth street, opposite the Capitol Grounds, at Richmond, and closed, with the fifth session, shortly after noon on Friday, September 19th. The hall, spacious without being too large, was well adapted for the purposes of the meeting. The exhibition was held in the supper-room, located in the high basement beneath the hall wherein the meeting was held, and was much more extensive than had been at first anticipated.

### FIRST SESSION—TUESDAY AFTERNOON.

Shortly after three o'clock the meeting was called to order by the President, Mr. Albert E. Ebert, of Chicago, who congratulated the members upon the happy auspices under which they were permitted to reassemble, and called attention to the pleasing coincidence that, at this moment, the British Pharmaceutical Conference was in session at Bradford, England, and the Australian Apothecaries' Association, at Vienna. He had this day received from the former the following kindly message :

BRADFORD, ENGLAND, September 16, 1873.

*To the President of the American Pharmaceutical Association, at Richmond, Virginia:*

Our members send hearty fraternal greetings to yours.

THE PRESIDENT

*Of the British Pharmaceutical Conference, at Bradford.*

To which he sent the following reply :

RICHMOND, VIRGINIA, September 16, 1874.

*To the President of the British Pharmaceutical Conference at Bradford, England:*

We return your fraternal greetings.

THE PRESIDENT
*Of the American Pharmaceutical Conference.*

The President then introduced to the meeting Hon. A. M. Keiley, Mayor of the City of Richmond, who, in a brilliant and happy speech, extended to the members of the Association and their ladies a most hearty welcome and cordial greeting. The frequent applause and, at the conclusion of the speech, the hearty cheers, testified how well his remarks had been appreciated by those present. The President replied briefly, expressing the thanks of the Association for the hospitable reception.

A Committee of Credentials was appointed by the chair, as follows: Prof. Procter, of Philadelphia; Geo. Leis, of Lawrence, Kansas; and Prof. G. F. H. Murkoe, of Boston; which Committee subsequently reported delegates from the following Associations, duly accredited to the meeting, viz.: the Massachusetts, New York, Philadelphia, Maryland, National (of Washington, D. C.), Louisville, Chicago, and Tennessee Colleges of Pharmacy, the New Jersey, Newark, Mississippi State, and East Saginaw Pharmaceuticial Association, the Alumni Association of the Massachusetts, New York, Philadelphia, and Maryland Colleges of Pharmacy, and the Literary and Scientific Society of the German Apothecaries of the City of New York.

At a subsequent session, the Secretary handed in the credentials of the Cincinnati College of Pharmacy, which had been previously overlooked. The credentials of the Kansas College of Pharmacy, and of the Alleghany County Pharmaceutical Association, not having arrived, Mr. Geo. Leis, of the former, and Messrs. W. H. Brill and H. S. Lutz, of the latter Society, were invited to seats as delegates.

Mr. Balluff, from the Business Committee, reported resolutions inviting to seats upon the floor, Professor Pratt, of Washington and Lee University, the members of the Faculty of the Virginia Medical College, and the Medical profession in general; also a resolution inviting the public to visit the exhibition on Wednesday evening, between the hours of 7½ and 9½ o'clock.

Dr. Meninger, on behalf of the Executive Committee, reported the names of 51 candidates for membership. Messrs. S. M. McCollin and E. C. Jones, being appointed tellers, the President was, on motion, directed to cast an affirmative ballot in favor of the candidates. An additional number of applicants for membership was soon after reported, and was likewise elected. At the first call of the roll 70 members were found to be present.

The following reports were handed in: Report of the Executive Committee, with the report of the Permanent Secretary of the Committees on the Progress of Pharmacy, on the Drug Market, on Papers and Queries, on Legislation, on Adulterations and Sophistications, on Arrangements for the Meeting of 1876, on Formulas of Elixirs, on the Editorship of the Reports on the Progress of Pharmacy, and on the Photographic Album.

The amendments to the by-laws proposed at the Cleveland meeting were, after some discussion, adopted. One of these amendments defines the Societies which shall be entitled to representation at the annual meetings to be *all local organizations of pharmacists* (Art. vi, Chap. vi); the other directs the appointment of the Committee on Specimens to be made at the first session, instead of at the second, as heretofore. The amendment to the Constitution, lying over from the previous meeting, and relating to the creation of a sinking fund, was for the present postponed.

An invitation from the Young Men's Christian Association to visit their rooms and make use of their library, was received and directed to be properly acknowledged by the Secretary.

Professor Proctor read the report of the Committee on the editorship of the Report on the Progress of Pharmacy. It recommends that the labor be entrusted to a salaried officer of the Association, to be called Reporter on the Progress of Pharmacy, proposes the necessary alterations of the Constitution and by-laws, and recommends the election of Professor C. L. Diehl, of Louisville, Kentucky, to this position. The report was accepted, and the proposed amendments adopted.

The following committee to nominate officers for the ensuing year was appointed by the delegations named above: Messrs. G. F. H. Markoe, David Hays, Chas. Bullock, Lewis Dohme, Wm. S. Thompson, C. L. Diehl, A. E. Ebert, B. Lillard, R. Rickey, C. W. Badger, J. T. Buck, S. S. Garrigues, C. A. Tufts, H. C. Porter, H. A. Vogelbach, A. A. Klienschmidt, Chas. Eimer,

Geo. Leis, and H. S. Lutz. To this number the following were added by the President from the Association at large: Messrs. A. Boyd, Utica, Ohio; Wm. Heyser, Chambersburg, Pennsylvania; A. S. Lee, Raleigh, North Carolina; William Vincent, Williamsburg, New York, and Charles M. Helman, Cincinnati, Ohio.

The reports of the Executive Committee and permanent Secretary were read. The former reports the decease of the following active and honorary members: Professor Edward Parrish, of Philadelphia; Kline C. Lineaweaver, of Washington, D. C.; T. W. Metcalf and W. E. Bayliss, of Brooklyn, New York; Elias Durand, of Philadelphia; Professor D. F. H. Ludwig, of Jena, and Dr. C. L. Arthur Casselmann, of St. Petersburg, Russia.

The report of the Secretary suggests that the Association grant permission to the Pharmaceutical journals of this country to print the essays and voluntary reports in advance of the publication of the proceedings. The incidental expenses during the year amounted to $402.53 of which sum $79.65 was for freight, and $143.50 for postage. An editorial note, appended to page 280 of the last volume of proceedings, was reported to be erroneous. The note which should be erased occurs in the description of the process for assaying sedlitz powders, as followed by Mr. C. W. Grassly, and is as follows:

The author does not state how he prevented the contamination of the precipitated sulphate of baryta with bitartrate of potassa, which must be precipitated after adding acetic and muriatic acids, except by *heating the resulting liquid.*

A letter from Mr. James R. Mercein, of Jersey City, one of the members of the Committee on the Progress of Pharmacy for the year 1871-72, was read, explaining that the portion of the annual report which he had agreed to furnish had been finished in due time, and that the chairman of that committee had been duly notified by him some time before the last meeting took place, but had failed to answer. It was then

*Resolved,* That Mr. Mercein be requested to present his report on the progress of pharmacy for the year 1871-72 to the Association.

Professor Diehl offered to arrange, if necessary, the report of Mr. Mercien for publication, which was accepted by resolution.

The Chair appointed the following Committee on Specimens:

Dr. A. W. Miller, of Philadelphia; M. L. M. Peixotto, of New York; Jos. L. Lemberger, of Lebanon, Pennsylvania; G. J. Luhn, of Charleston, South Carolina, and David Hays, of New York. At the next session, Mr. Peixotto was excused from serving on this committee, and Mr. W. H. Scott, of Richmond, appointed in his place.

The Secretary laid before the Association two volumes of the Medical and Surgical History of the War of the Rebellion, received from the Surgeon-General of the United States Army, also one volume of the Report of Columbia Hospital for Women, and Lying-In Asylum, Washington, D. C., by J. Harry Thompson, A.M., M.D.

The President read his annual address, reviewing the labor of the committees, and making several important recommendations, aiming at an increased usefulness of the Association. The address was well received, and referred to a committee of three, consisting of Messrs. Charles Bullock, L. V. Heydenreich and Dr. E. P. Nichols. The Association then adjourned until 9 o'clock the following morning.

SECOND SESSION—WEDNESDAY MORNING.

After the reading and approval of the minutes of the first session, the Nominating Committee recommended the election of the following officers for the ensuing year:

*President*—John F. Hancock, of Baltimore, Maryland.

*Vice Presidents*—William Saunders, of London, Ontario; John T. Buck, of Jackson, Mississippi, and Paul Balluff, of New York.

*Treasurer*—Charles A. Tufts, of Dover, New Hampshire.

*Permanent Secretary*—John M. Maisch, of Philadelphia.

*Reporter on the Progress of Pharmacy*—C. Lewis Diehl, of Louisville, Kentucky.

*Executive Committee*—Thomas S. Wiegard, chairman, of Philadelphia; George Leis, of Lawrence, Kansas; Chas. L. Eberle, of Philadelphia; Henry J. Menninger, of Raleigh, North Carolina, and John M. Maisch, *ex officio.*

*Committee on Drug Market*—P. W. Bedford, chairman, of New York; Wm. H. Brown, of Baltimore; Wm. P. Keffer, M.D., of New Orleans; William H. Brill, of Pittsburg, and William S. Merrell, of Cincinnati.

*Committee on Papers and Queries*—Jos. P. Remington, chairman, of Philadelphia; Lewis Dohme, of Baltimore, and Benj. Lillard, of Nashville, Tennessee.

*Business Committee*—Edward P. Nichols, M.D., chairman, of Newark, New Jersey; Joel S. Orme, of Cambridgeport, Massachusetts, and John F. Judge, of Cincinnati.

The report was accepted, and Messrs. Boyd and Hassencamp having been appointed tellers, the President was directed to deposit an affirmative ballot for all the nominees. Professors Procter and Stabler were appointed a committee to conduct the President elect to the chair; but Mr. John F. Hancock not being in the hall, the first Vice President elect, Mr. Wm. Saunders, was invited to preside.

Dr. Tufts read the annual report of the Treasurer, which was accepted, and referred to an Auditing Committee, to be appointed by the President.

There was a balance of over $900 in the hands of the Treasurer at the beginning of the meeting.

The following letter was read:

SEPTEMBER 12, 1873.

*To the American Pharmaceutical Association:*

*Gentlemen,*—To further original investigation, and to inaugurate a system of prizes by this Association to those who, by study and application, shall add to our knowledge of medicinal substances, I have the pleasure to present to this Association the inclosed sum ($500) to be used in the following manner: The money to be properly invested by order of the Executive Committee, and the annual interest derived therefrom to be appropriated for conferring a suitable prize for the best essay or written contribution containing an original investigation of a medical substance, determining new properties or containing other meritorious contributions to knowledge, or for improved methods of determined merit for the preparation of chemical or pharmacal products. The prize to be awarded by a suitable committee within six months after the annual meeting at which the essays are presented for competition; *Provided* that, in case no one of the essays offered is of sufficient merit to justify the award, in the judgment of the committee, all may be rejected, and the sum added to that of the fund.

Respectfully, ALBERT E. EBERT.

The gift was accepted with the thanks of the Association, and the President directed to appoint a committee of three to carry out the objects of the donor. This fund was, later in the session, directed to be called the "Ebert Fund," and the prizes awarded from the proceeds thereof, the "Ebert Prize," agreeable to a resolution offered by G. Leis.

The President elect having arrived, took the chair after some appropriate remarks, and appointed Messrs. N. H. Jennings, of Baltimore; Benjamin Stacy, of Cambridgeport, and T. Roberts Baker, of Richmond, a committee to audit the Treasurer's accounts.

Propositions for membership were received, and the applicacants duly elected, Messrs. Hassencamp and Jones acting as tellers.

The report of the Committee on Adulteration and Sophistications was read by Mr. Peixotto, accepted and referred.

The report on the Progress of Pharmacy, read by Professor Diehl, was ordered to take the same course. On motion, the sum of $250 was directed to be paid to Professor Diehl, as a partial acknowledgment for his time and labor in voluntarily preparing this report.

Letters were read from Mr. W. G. R. Frayser and Messrs. C. R. Rees & Co., offering to prepare a photographic group picture of the members of the Association. The Secretary was directed to acknowledge both letters and inform the writers that their kind offers must be respectfully declined for want of time.

Prof. Diehl read the report of the Committee on Arrangements for the meeting of 1876, which, after suggesting various measures for the celebration, recommends that the meeting of 1875 be held in the city of Boston, in order to have a full attendance, and the better to arrange for the centennial.

The resignation of Mr. C. W. Grassly, of Chicago, was offered and read. This communication being deemed insulting to the officers and members of the Association, his resignation was rejected, and, on motion of Dr. Squibb, he was expelled, by a vote of 58 to 2, for using indecorous language to the Association and its officers.

Dr. Squibb read a volunteer paper on the buying and selling of alcohol, which was accepted and referred.

A Committee of three, on the time and place of the next annual meeting, was directed to be appointed. The President named Messrs. Joel S. Orme, R. H. Gardner and Louis Dohme to serve on this Committee.

The Association then went in a body to the exposition room, and afterwards adjourned until 3 o'clock, P.M

THIRD SESSION—WEDNESDAY AFTERNOON.

The minutes of the previous session having been read and approved, the report of the Auditing Committee was read and accepted. The Committee found the accounts correct, and the books in admirable order.

The salary of the Reporter on the Progress of Pharmacy was next considered, and, on motion of Mr. Peixotto, seconded by Dr. Squibb, the By-Laws were so amended as to provide that he shall receive such sum for his report as shall be annually determined.

On motion, the regular order of business was postponed till the following morning, and this afternoon session mainly devoted to the reading and discussion of papers.

Mr. H. N. Rittenhouse read a paper in answer to query 1, on a permanently flexible isinglass plaster. Mr. P. F. Lehlbach, in answer to No. 7, presented a paper on *Sapo mollis*, accompanied by some samples.

Dr. Squibb read a paper on rhubarb, and called particular attention to the fact that even the best rhubarb of commerce appeared to be losing that peculiar aromatic odor formerly so noticeable, and which he considered one of the most reliable tests of the root. He accounted for this by suggesting that artificial process of drying, forced production, etc., might be the causes. He submitted three boxes of specimens of the best chests that had been imported at New York the past year. On motion, this was accepted and referred for publication.

Dr. Squibb also read an essay on Physicians' Pocket-Cases, in which he described one of his own contrivance, provided with a minim liquid measure, or pipette, to draw the contents from the vials without dropping or pouring, and with more accuracy than by either of those processes. He presented specimens of the cases, &c., and announced that they could be made and used by any one who chose, as he did not propose to patent them.

The following letter was read by the Secretary:

RICHMOND, VA., Sept. 17, 1873.

*To the Officers and Members of the American Pharmaceutical Association:*

GENTLEMEN,—In behalf of the Pharmacists and Druggists of this city, I hereby extend to you a cordial invitation to participate with us in an excursion down James River, on the afternoon of Thursday, 18th inst., at 3 o'clock.

Very respectfully,

WM. H. SCOTT, *Ch'n Committee of Arrangements.*

Prof. Markœ, one of the members of the Pharmacopœia Committee, read an individual report, containing criticisms on the pharmacopœia, which was accepted and referred.

In answer to query 10, Mr. Bedford reported verbally that balsam of tolu cannot be emulsionized.

A partial answer to query 12, on the use of petroleum benzin for extracting oleoresinous drugs, was read by J. P. Remington, and the subject continued to him for another year.

A paper, by Mr. E. D. Chipman, on the proper proportions of sugar and honey in Vallet's mass, was read in answer to query 15, also one by Prof. Proctor, on the value of orange-colored glass as a preventive of the chemical influence of light; this subject was continued for another year.

The following papers were read: by the Secretary, on query 21, in relation to the proper time for collecting biennial medical plants; by J. P. Remington, on cheap ointment boxes; by Prof. Proctor, on query 24, on the preparation of cucumber ointment; by Dr. Pile, on improvements in graduated measures, and on query 28, on labelling shop furniture, etc., by G. H. Schafer.

In a voluntary paper, Mr. G. W. Kennedy informs that he has separated gentistic acid and gentiopierin from columbo (*Frasera Walteri.*)

Several new members were elected, Messrs. Kennedy and Lemberger acting as tellers.

The following letter was read by the Secretary:

RICHMOND, September 16, 1873.

*Prof. J. M. Maisch, Permanent Secretary of the American Pharmaceutical Association, in session at Richmond, Va.:*

DEAR SIR,—It affords me pleasure to be in position to offer an exchange of our Society's publications as a partial expression of our high appreciation of the laudable objects for which your Association is organized. You may feel assured that whatever you may do to elevate the standard of your profession and thereby discountenance quackery and dishonest imposition, will meet with the hearty favor and cordial support of the community in which you are assembled, and especially of the Medical Society of Virginia.

Very respectfully, yours, etc.,

LANDON B. EDWARDS,

*Recording Secretary Medical Society of Virginia.*

On motion, the proposed exchange was accepted, and the Secretary directed to notify Dr. Edwards.

The Association now adjourned until 9 o'clock the following morning.

FOURTH SESSION—THURSDAY MORNING.

After the minutes had been read and approved, Dr. Nichols presented the report of the committee appointed at the first session, to consider the suggestions contained in the President's address and Secretary's report.

Some discussion ensued upon the recommendation of the report that the Executive Committee and Permanent Secretary be allowed a discretion in the publication in pharmaceutical journals, or elsewhere, of any essay or article read before the Association in advance of the publication of the annual volume of transactions. Dr. Squibb moved to strike it out. Agreed to. The report, as amended, was then adopted.

The proposed amendment to the Constitution to provide for a sinking fund, offered last year, was rejected.

After some discussion on the propriety of declining, in advance, for future meetings, all invitations to excursions until the labors of the Association shall have been performed, the committee on the next annual meeting presented their report, recommending the Twenty-second Annual Meeting to be held in the city of Louisville, on the second Tuesday in September, 1874. The report was received and adopted without a dissenting vote, and Prof. Emil Scheffer was, by acclamation, elected Local Secretary.

The reports of the Committees on Legislation and on the Photographic Album were read and the Committees discharged. The report of the Committee on Drug Market was read by Mr. Bedford, accepted and referred.

The President appointed the following Committee on Album: P. W. Bedford, of New York, Chairman; C. A. Tufts, of New Hampshire; R. J. Brown, of Kansas; J. P. Remington, of Philadelphia; E. H. Sargent, of Chicago.

The President also announced the appointment of the following Committee on "Ebert prize:" Messrs. Charles Bullock, Wilson H. Pile and John M. Maisch, of Philadelphia.

Prof. Proctor read a valuable essay on query 30, "What shall I read, and where shall I begin?" Prof. Lillard, a voluntary paper on homœopathic pharmacy, and Dr. Garrigues on American bromine production. The latter also exhibited oak galls collected by him near Huntington, W. Va. Dr. Squibb stated that galls from the oak and sumach had been used for making gallic acid.

The following papers were read: On poisons and the protection against their improper use, by C. L. Eberle; on the insect enemies of drugs, and on the Mexican honey ant, by William Sanders; on a general apparatus stand, etc., and on ergot and its preparations, by Dr. Squibb; on the proper alcohol strength of tincture of colombo, by C. L. Eberle; on the purity of commercial tartaric acid, by H. J. Rose. After some verbal remarks on under and over-hydrated chloral, by Prof. Diehl, query 34 was dropped.

Mr. J. F. Hancock read the report of the Committee on Elixirs, etc., which was accepted and the committee discharged. It was then

*Resolved*, That this report be adopted, with the recommendation that these formulas be used by members of the Association, and that the Secretary be instructed to send a printed copy of this report to the Medical Societies of the Union, with the suggestion that physicians, if prescribing elixirs at all, prescribe only such formulas as have been adopted by this Association.

*Resolved*, That Mr. J. F. Hancock be appointed the Committee on Unofficial formulas.

The delegates of all incorporated pharmaceutical societies not represented on the Committee on the Pharmacopœia, were requested to nominate one member for said Committee, and report at next session. After the election of new members, Messrs. R. W. Gardner and C. W. Badger having acted as tellers, the Committees on Adulterations and Sophistications, on Legislation, on Liquor Dealers' License of Apothecaries, and on Infringement of Stamp Tax, were continued for another year, Dr. E. R. Squibb having, at his request, been previously excused from serving on the Committee on Liquor Dealers' License, and his name having been substituted by that of R. W. Gardner, of Jersey City.

A vote of thanks to the retiring officers was passed, and the Association adjourned until Friday morning at 9 o'clock.

FIFTH SESSION—FRIDAY MORNING.

After the reading and approval of the minutes, the amendment of the By-Laws was adopted, requiring the President to discuss, in his Annual Address, such scientific topics as he may select, instead of reviewing the labors of the Committee during the past year.

The report of the Committee on Specimens was read by Dr. A. W. Miller, accepted and referred.

On motion of Dr. Squibb, the salary of the reporter on Progress of Pharmacy was fixed at $400 for the ensuing year. Notice was also given by Dr. Squibb of a proposed amendment of the By-Laws to increase the salaries of the Treasurer and Permanent Secretary $100 per annum, in view of the increased duties of both officers. The proposition lies over until the next meeting.

The following essays were read and referred: On the amount of carbolic acid necessary to prevent the growth of organism in solutions of alkaloids, by E. H. Squibb; on the influence of heat upon the medicinal properties of sarsaparilla, by Prof. J. F. Judge; on the reaction of chloral hydrate, by J. M. Maisch; on gelseminia and gelseminic acid, by C. F. Fredigke; on assaying cantharides and their preparations, by J. M. Maisch; on Indian remedies, by B. Stacey; on weights and measures, by Prof. O. Oldberg; on fluid extract of vanilla, by Dr. E. P. Nichols; on fluid extract of sarsaparilla, by Prof. Judge; also a paper by A. T. Moith, discussing the relations of physicians and apothecaries.

The Secretary exhibited a sample of muriate of cinchonia, which is put up in New York in fraudulent imitation of sulphate of quinia of French manufacture, and at the present time extensively sold into the interior towns of the Southern States.

Prof. Markoe showed diatomaceous earth, collected by him in Richmond, Va.

After the election of new members, Messrs. Remington and Kennedy acting as tellers, the Business Committee offered the following:

The American Pharmaceutical Association is now about to close its Twenty-first Annual Session in the capital of the Old Dominion. Some hesitation was felt at the last meeting in deciding on this place, but the success of our experiment at Cleveland induced us to repeat it. The kind courtesy and generosity of our reception, the abounding old Virginia hospitality of the pharmaceutists and citizens of Richmond, and the beautifully appropriate welcome of His Honor the Mayor of this city, has dispelled every doubt of the propriety of our selection. Nothing has been left undone that could add to our comfort and pleasure; and when we leave this place to return to our homes, we shall carry with us pleasant recollections of this visit which will not soon be effaced from our memory. As a faint expression of our warm appreciation of the kindness shown us, be it

*Resolved*, That the hearty thanks of this Association be and are hereby tendered to the Pharmaceutists and citizens of Richmond for the cordiality of our reception at this our first visit to the "Sunny South."

President Hancock arose, and in a few appropriate and well-timed remarks, bore testimony to the warmth of the reception of the Association at the hands of the pharmaceutists and citizens of this city. He said that they had never met with a heartier reception anywhere. He indulged the hope that at their next meeting, in Louisville, representatives would be present from the Virginia Pharmaceutical Association.

Thanks were also tendered the Richmond press for their faithful reports of its proceedings; after which the Secretary, at 12¼ o'clock, offered a resolution, which was adopted, that the Association do now adjourn, to meet on the second Tuesday in September next, at 3 o'clock P.M., at Louisville, Ky.—*American Journal of Pharmacy*.

